# Ensuring the Safety of Yellow Fever Vaccination in Travelers—The Experience at a Large U.S. Academic Medical Center in Colorado

**DOI:** 10.3390/tropicalmed5030125

**Published:** 2020-07-29

**Authors:** Mehdi Bandali, Jonathan Schultz, Kimlien Than, Donna McGregor, Solana Archuleta, Sindhu Chadalawada, William Mundo, Daniel Chastain, Carlos Franco-Paredes, Elaine Reno, Andrés F. Henao-Martínez

**Affiliations:** 1 School of Medicine, University of Colorado Anschutz Medical Campus, Aurora, CO 80045, USA; mohamed.bandali@cuanschutz.edu (M.B.); solana.archuleta@cuanschutz.edu (S.A.); william.mundo@cuanschutz.edu (W.M.); 2 Division of Infectious Diseases, Department of Medicine, University of Colorado Anschutz Medical Campus, Aurora, CO 80045, USA; jonathan.schultz@cuanschutz.edu (J.S.); donna.mcgregor@cuanschutz.edu (D.M.); andres.henaomartinez@cuanschutz.edu (A.F.H.-M.); 3 School of Pharmacy, University of Colorado Anschutz Medical Campus, Aurora, CO 80045, USA; kimlien.than@cuanschutz.edu; 4 NRI General Hospital, Pradesh 522503, India; chsindhu@gmail.com; 5 Department of Clinical and Administrative Pharmacy, University of Georgia College of Pharmacy, Albany, GA 30901, USA; daniel.chastain@uga.edu; 6 Department of Emergency Medicine, Hospital Infantil de México, Federico Gómez, México City 06720, Mexico; elaine.reno@cuanschutz.edu

**Keywords:** yellow fever virus, yellow fever vaccine, travel medicine, health policy

## Abstract

**Background:** Yellow fever (YF) virus has the potential to cause fatal outcomes among at-risk individuals visiting endemic areas. Vaccinating travelers who are at risk is necessary to prevent virus-related life-threatening complications. We lack data on the clinical features of persons seeking YF vaccination. We aim to describe the characteristics of a cohort of persons receiving the YF vaccine before travel. **Methods:** A retrospective analysis of 964 travelers receiving the YF vaccine (Stamaril^®^) from Oct 2016 to Jul 2019 was performed at the University of Colorado Hospital, U.S. Percentages, means, and standard deviations were calculated. A multivariate logistic regression model was built to evaluate the association between receiving YF vaccination less than 10 days before departure and visiting friends and relatives (VFR). **Results:** The average age of the subjects was 39 ± 18 years with a range of nine months to 83 years. Persons who were 60 years of age and older represented 17%. Women consisted of 52%, and most of the travelers were Caucasians (64%). Travelers reported traveling to Africa (57%) or South America (40%). The primary destinations for travelers overall were Kenya (19%), Uganda (11%), and Tanzania (11%) in Africa; and Peru (14%) and Brazil (13%) in South America. The most common reasons for travel included leisure (44%), VFR (18%), and mission trips (10%). Comorbidities included a history of hematologic disorders (4%), HIV infection (2%), and diabetes mellitus (3%). The average duration between vaccine administration and travel was 43 days. Those VFR were two times more likely to receive the YF vaccination <10 days before departure. **Conclusions:** Identifying the type of travel, itinerary, and underlying medical conditions allows providers to administer the YF vaccine to travelers safely. There is a need to identify strategies to improve the timing of YF vaccination among VFR travelers.

## 1. Introduction

Yellow fever (YF) is a hemorrhagic disease caused by a flavivirus and transmitted by the *Aedes aegypti* mosquito, which is found in parts of tropical South America and sub-Saharan Africa. Since the early 1990s, the World Health Organization (WHO) estimates that there have been 200,000 cases of YF and 30,000 deaths due to the disease worldwide [1]. An analysis of African data sources in 2013 estimated that the burden of YF was 130,000 severe cases and 78,000 deaths [2]. 

Treatment for patients with YF is mainly supportive, as there is no specific antiviral therapy available. However, the YF vaccine is widely used for the prevention of YF in travelers, and for people living in endemic areas. There are 20–60 million doses of the vaccine distributed annually [3]. YF-VAX^®^ and Stamaril^®^ are live attenuated vaccines prepared by culturing the 17D-204 strain of the virus in chicken embryos, and their efficacy is based upon the development of neutralizing antibodies [4]. The vaccine has been used since the 1930s when it was first developed. WHO modified the length of validation by the vaccine in 2016 from 10 years to lifelong duration for most individuals [5]. In the U.S., the YF vaccine is primarily given as prophylaxis to military personnel and patients at risk due to travel to endemic areas. Sanofi held the manufacturing of YF-VAX^®^ in 2016 due to factory production issues, and the company made available an alternative vaccine, Stamaril^®^, which is administered in the U.S. through an FDA-approved expanded access program. Standard clinical practice of screening patients during the pre-travel encounter is essential to prevent complications associated with YF vaccination. YF is a live attenuated vaccine with known serious adverse events, including vaccine-associated viscerotropic disease (YEL-AVD) and neurotropic disease (YEL-AND). Risk factors include uncontrolled HIV infection, 60 years and older patients [6], and other immunocompromised conditions. The subgroup of patients traveling to visit friends and relatives (VFR) can be a particularly vulnerable population at higher risk for some preventable infections, such as malaria, due to loss of previous immunity, and a lesser likelihood to seek pre-travel advice or take prophylaxis, while going back to their home countries [7,8]. There are limited studies in the U.S. that describe the patient population receiving the YF vaccine. This study aimed to identify important descriptors of patients getting the YF vaccine before travel, including patients traveling to visit friends and relatives. This information can aid public health agencies to enhance strategies to increase immunizations of patients at risk and avoid complications.

## 2. Methods

### 2.1. Ethics Statement

The present investigation complies with the Health Insurance Portability and Accountability Act (HIPAA) according to the Colorado Multiple Institutional Review Board (COMIRB) at the University of Colorado Denver. Patients received the YF vaccine through an approved Sanofi Pasteur Inc. Protocol Number STA00011, Expanded Access IND Program to Provide Stamaril^®^ YF Vaccine (17D-204 strain) to Persons in the United States (Quorum Review File #32032). Analysis of clinical data has been performed under an approved protocol (COMIRB Protocol 17-1032).

### 2.2. Patients and Data Collection

Data from patients receiving the Stamaril^®^ vaccine at the University of Colorado Hospital clinic from 31 October 2016 to 7 July 2019, were submitted for data extraction. Electronic medical records (EPIC) were automatically interrogated for the cohort of travelers through a software supported by Health Data Compass Data Warehouse project (healthdatacompass.org). Study data were collected and managed using REDCap electronic data capture tools hosted at the University of Colorado Denver. The following variables were automatically collected: gender, age, race, state of residency, date of YF vaccine, and the following comorbidities based on International Classification of Diseases (ICD) codes: diabetes mellitus, neoplasms, HIV infection, history of hematology-immune disorders, and pregnancy (see Appendix A for full ICD-9, and ICD-10 code definitions). VFR was defined as a form of travel wherein the purpose of the trip or the type of accommodation was visiting friends and/or relatives as consigned in the patient’s history. The following variables were manually collected through chart review: verification of vaccine date if it was unavailable per the automatic search, pregnancy at the time of vaccine administration, the reason for travel, the continent of travel, and destination countries. Some travelers had missing information on key variables (Table 1).

### 2.3. Statistical Analysis

The means and standard deviations for continuous variables were calculated. For categorical variables, frequencies and percentages were calculated. Patient characteristics were compared between those reporting to visit friends and relatives versus other reasons of travel using chi-squared, students *t*-tests, or Fisher exact tests. A multivariate logistic regression model was built to evaluate the association between those receiving YF vaccination less than 10 days before departure and those VFR, after controlling for confounders of age, sex, race, and the continent of travel. All analyses were conducted in SAS 9.4. We selected the 10 days based on WHO recommendation of the optimal time of immunization against YF before traveling to endemic areas [9].

Data access: The corresponding author had full access to all the data in the study and had final responsibility for the decision to submit the manuscript for publication. The datasets generated during and/or analyzed during the current study are available from the corresponding author upon reasonable request.

## 3. Results

### 3.1. Clinical Characteristics of Patients Receiving the Yellow Fever Vaccine:

**Figure 1:**
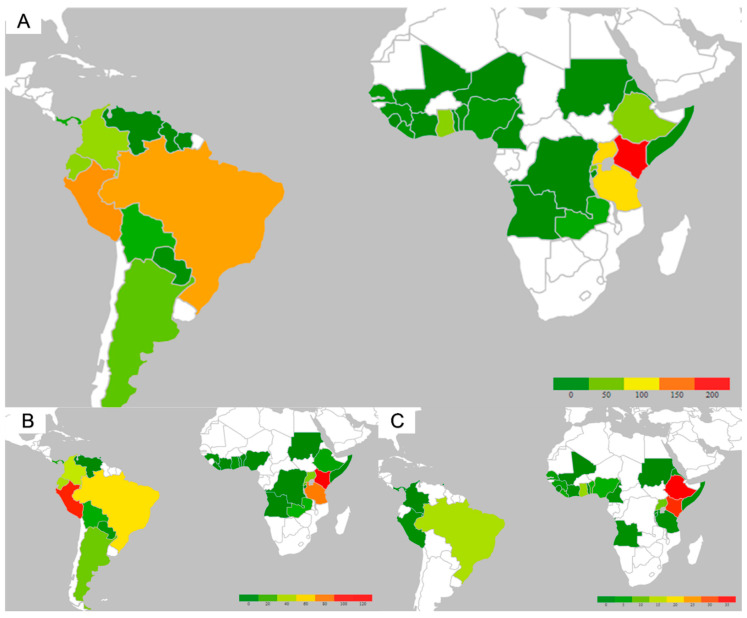
Heat map of destinations among patients receiving the yellow fever (YF) vaccine. (**A**). Heat map of countries of destination for the total cohort. (**B**). Heat map for travelers going for leisure. (**C**). Heat map for travelers going to visit friends and family. The density of patients is represented by color bars.

**Figure 2:**
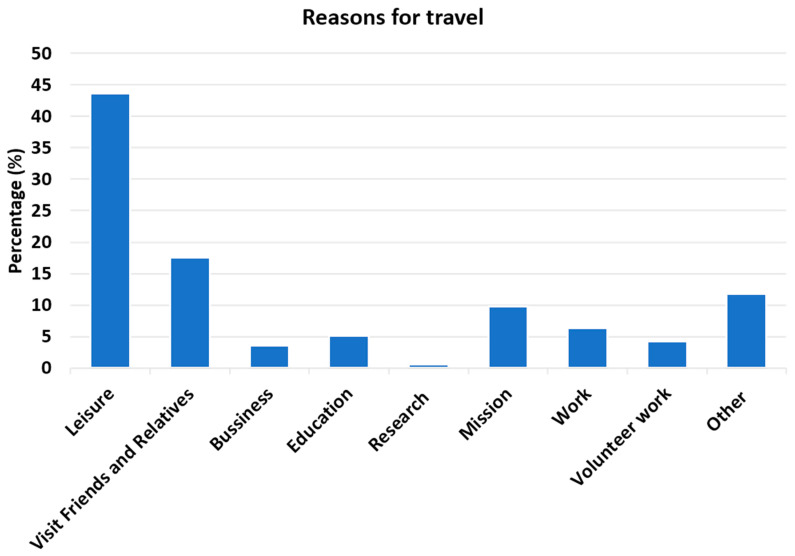
Reasons for travel among patients receiving the YF vaccine (n = 964).

Of 964 subjects, the average age of travelers receiving the vaccine was 39 years with a range from nine months to 83 years (Table 1). There were more females (52%), and most of the travelers were identified as Caucasian (64%). Most travelers were from the State of Colorado (96%). Travelers predominantly reported travel to Africa (57%) or South America (40%), among which the primary destinations included Kenya (19%), Uganda (11%), and Tanzania (11%) in Africa; and Peru (14%) and Brazil (13%) in South America (Figure 1). The most common reasons for travel included leisure (44%), followed by VFR (18%) and mission trips (10%) (Figure 2). Comorbidities were uncommon but included a history of neoplasm (7%), hematologic/immunologic disorders (4%), HIV infection (2%), and diabetes mellitus (3%). Heme/immune diagnosis captured through ICD codes included benign heme disorders such as polycythemia, pancytopenia, sickle cell trait, thalassemia, history of deep venous thrombosis, previous use of systemic lupus erythematous medications, and unspecified immune disorders (Appendix A). Common ICD neoplasm diagnoses captured were benign tumors of the skin, prostate, and uterus; and history of colorectal cancer, melanoma, multiple myeloma, uterus carcinoma, kidney cancer, bladder cancer, and others (Appendix A). The average duration between vaccine administration and travel was 43 days. No evidence of mild or life-threatening reactions to Stamaril^®^ occurred in this large cohort.

Travelers who were 60 years of age and older represented 17% of the total study population. They were 53% women, predominantly white (79%), and mostly traveling for leisure (63%). They visited Africa (52%) and South America (47%) more often and had plenty of time to receive the vaccine before departure with a mean of 50 days. Kenya (23%), Tanzania (22%), Brazil (24%), and Peru (16%) were popular destinations among these travelers. Only 7% received the vaccine less than 10 days from departure. Sixty years or older travelers had higher rates history of heme-immune conditions (8%), diabetes mellitus (8%), and neoplasms (22%).

Since the vaccine is licensed only to infants older than nine months of age, we only had four infants less than one year of age in our cohort, representing 0.4%. We did not have any reported side effects or complications in this group of travelers.

### 3.2. Travelers Visiting Friends and Relatives

Travelers visiting their friends and relatives were more predominantly men (56%), younger, and identified as African Americans for their primary ethnicity. Women in this subgroup were more likely to be pregnant and more likely to be HIV positive, but less likely to have a history of cancer. They were more likely to visit Africa as opposed to South America, and they had less time between vaccine administration and travel departure. Common destinations were Kenya, Ethiopia, and less commonly Brazil (Figure 1C). VFR travelers were more likely to receive the vaccine less than 10 days before departure compared to other reasons for travel (18% vs. 10%, *p* = 0.005). Those VFR were 2.2 times (OR 2.2 (1.3–3.7), *p* = 0.003) more likely to receive the YF vaccination <10 days before departure, after controlling for confounders of age, sex, race, and destination of Africa vs. South America.

## 4. Discussion

We describe the clinical characteristics of a cohort of travelers seeking YF vaccination at a U.S medical center. Some of those travelers presented themselves as family groups. Most were young adults, but the age varied widely from infants to seniors. In Colorado, most travelers were Caucasian, had a few comorbidities, and traveled to Africa most often. Travelers sought YF vaccination on an average of about a month and a half before their departure date. The subgroup of VFR travelers was younger, of African American descent, traveling to the African continent, more often pregnant, and had a higher likelihood of having an HIV infection.

We also showed that VFR travelers were more likely to receive the YF vaccine at a suboptimal time before travel. WHO recommends immunization against YF at least 10 days before travel to endemic areas [9]. Studies in travel clinics have shown inadequate timing of the YF vaccine before travel in children [10]. Those VFR may have a harder time making travel clinic appointments and may present just before travel and may be less prepared to take appropriate preventative measures [11].

Receiving the vaccine less than 10 days prior can also have implications for possible denial of entry or increased paperwork at the country of destination. Those VFR may also not recognize the specific country requirement of YF vaccination until just shortly before departure. VFR travelers carry a higher risk of acquired travel-related illnesses such as Hepatitis A, typhoid, malaria, soil-transmitted helminths, and influenza [12]. Specific risk factors associated with the increased threat of illness among VFR include longer stays, decreased pre-travel health plans, sick contacts while abroad, and poorer sanitary conditions during their stay. Public health interventions can aim to increase rates and enhance the optimal timing of YF vaccination among those VFR.

We have shown a large cohort of travelers who safely received the YF vaccine before travel. Since the rate of adverse events with the Yellow vaccine is of about three events per 100,000 doses [13], with our relatively small representative sample we cannot extrapolate a different safety profile. Nevertheless, through standardized travel advice encounters, we safely delivered the vaccine to more than 150 travelers aged 60 years or older, travelers with controlled HIV, and history of cancer, pregnancy, or heme-immune disorders not listed as absolute contraindications. Although travel clinic providers screened travelers for contraindications to receive the YF vaccine, the more comprehensive interrogation of our electronic medical records found a small rate of non-prohibitive relative contraindications in some travelers. We still encourage the avoidance of vaccination in travelers with relative contraindications if the risk of YF acquisition during travel is deemed low, but our findings suggest vaccination is safe in this relatively small cohort among travelers older than 60 years of age. Our cohort delivered some safety evidence of YF vaccine administration among travelers with those listed conditions. This data can reassure clinicians and travelers with a history of those conditions to make pre-travel decisions where the YF vaccine administration is mandatory.

Previous reports in Nigeria have documented the safe administration of the YF vaccine during pregnancy [14]. Administration of the vaccine to HIV-infected individuals with CD4 counts greater than 500 cells/mm^3^ is safe [15]. YF vaccine has been also administered safely in immunocompromised patients after the withdrawal of their immunosuppressive therapy [16,17].

Our population of travelers visiting family and friends reflects the diversity of African immigrants in Aurora, Colorado. A significant number of travelers seeking pre-travel advice will benefit from a continued comprehensive pre-travel screening of immunocompromised conditions.

Providing the YF vaccine remains a critical public health strategy to decrease transmission and disease. Although overall coverage for the YF vaccine has increased in endemic countries [18], travelers are an important target for this preventive strategy as well. Data from the recent Brazilian outbreak found a case fatality of up to 40% among unimmunized travelers [19].

Models incorporating clinical features have been important to showcase disease burden and to enhance vaccination strategies [20]. The study of high-risk populations can inform the best vaccine policies [21]. VFR travelers are considered a high-risk population. We recommend public health policies to enhance the inclusion of vulnerable populations such as people VFR for YF prophylaxis. We should explore policies such as outreach community messages on the importance of pre-travel health care among foreign-born populations in the US. Additional considerations include community health workers reaching VFR communities to explain the importance of pre-travel vaccination and assessing individual risks.

There are a few limitations to this study. The retrospective selection of data limits the reliability and number of variables analyzed. Misdiagnosis or irrelevant past medical history could have been selected through the automatic ICD screening of the previous diagnosis. However, selection bias was decreased through the automatic collection of some key risk factor variables. Missing data occurred in some medical records as well. We did not have data on foreign-born status among the VFR travelers, which can also account for different clinical characteristics or outcomes.

YF vaccine remains a priority for decreasing disease burden. Coloradans seeking the vaccine represent the current demographics of our community. Despite the history of uncommon well-known comorbidities, YF vaccination was effective and safe. YF disease remains a potentially lethal complication during travel. The current outbreak in Nigeria and the 2018 outbreak in Brazil, both with high case fatality ratios, highlight that prevention strategies are a priority. Efforts should be enhanced to continue YF disease prevention strategies in travel clinics throughout the United States.

## Figures and Tables

**Table 1:** A cohort of travelers receiving the yellow fever vaccine at the University of Colorado Hospital.

Variables	Total (n = 964)	Visit F&R, N = 170 (18%)	Other Reason, N = 794 (82%)	*p*-Value
Age (years), mean (SD)	39 (18)	30 (20)	41 (17)	<0.0001
Age ≥ 60 years old	167 (17%)	18 (11%)	149 (19%)	0.01
Sex, female	502 (52%)	74 (44%)	428 (54%)	0.01
Race				<0.0001
White	614 (64%)	36 (21%)	578 (73%)	
African American	142 (15%)	104 (61%)	38 (5%)	
Other	208 (22%)	30 (18%)	178 (22%)	
Out of Colorado State	41 (4%)	5 (3%)	36 (5%)	0.35
Pregnancy	11 (2%)	5 (6%)	6 (1%)	0.004
Hematologic/Immunulogic Disease	36 (4%)	9 (5%)	27 (3%)	0.237
Diabetes Mellitus	27 (3%)	6 (4%)	21 (3%)	0.526
Neoplasm	72 (7%)	7 (4%)	65 (8%)	0.07
HIV	22 (2%)	12 (7%)	10 (1%)	<0.0001
Destination				
Africa	551 (57%)	146 (86%)	405 (51%)	<0.0001
South America	387 (40%)	22 (13%)	365 (46%)	
Other	26 (3%)	2 (1%)	24 (3%)	
Time between vaccine administration and depature (days), mean (SD)	41 (38)	34 (39)	43 (38)	0.0049
Vaccination < 10 days	110 (11%)	30 (18%)	80 (10%)	0.005

## Appendix A

**Table A1:** List of diagnoses by ICD codes in yellow fever recipients, University of Colorado Hospital, Denver, 2016–2019.

ICD	ICD-Code	Diagnosis	Categories
ICD-9-CM	250.8	Xanthoma diabeticorum	DM
ICD-9-CM	250.6	Well controlled type 2 diabetes mellitus with peripheral neuropathy (HC code)	DM
ICD-9-CM	250.7	Well controlled type 2 diabetes mellitus with peripheral circulatory disorder (HC code)	DM
ICD-9-CM	250	Well controlled type 2 diabetes mellitus (HC code)	DM
ICD-9-CM	250.61	Well controlled type 1 diabetes mellitus with peripheral neuropathy (HC code)	DM
ICD-9-CM	250.01	Well controlled type 1 diabetes mellitus (HC code)	DM
ICD-9-CM	250.5	Visual loss due to diabetes mellitus (HC code)	DM
ICD-9-CM	250.9	Unspecified diabetes mellitus with unspecified complications	DM
ICD-9-CM	250.02	Uncontrolled type II diabetes mellitus with nephropathy	DM
ICD-9-CM	250.92	Uncontrolled type 2 diabetes mellitus with complication (HC code)	DM
ICD-9-CM	249.6	Ulnar neuropathy due to secondary DM (HC code)	DM
ICD-9-CM	250.81	Type I diabetes with complications	DM
ICD-10-CM	E11.9	Type 2 diabetes mellitus without complications	DM
ICD-10-CM	E11.311	Type 2 diabetes mellitus with unspecified diabetic retinopathy with macular edema	DM
ICD-10-CM	E11.8	Type 2 diabetes mellitus with unspecified complications	DM
ICD-10-CM	E11.69	Type 2 diabetes mellitus with other specified complication	DM
ICD-10-CM	E11.39	Type 2 diabetes mellitus with other diabetic ophthalmic complication	DM
ICD-10-CM	E11.49	Type 2 diabetes mellitus with other diabetic neurological complication	DM
ICD-10-CM	E11.59	Type 2 diabetes mellitus with oth circulatory complications	DM
ICD-10-CM	E11.3393	Type 2 diabetes mellitus with moderate nonproliferative diabetic retinopathy without macular edema, bilateral	DM
ICD-10-CM	E11.3293	Type 2 diabetes mellitus with mild nonproliferative diabetic retinopathy without macular edema, bilateral	DM
ICD-10-CM	E11.3219	Type 2 diabetes mellitus with mild nonproliferative diabetic retinopathy with macular edema, unspecified eye	DM
ICD-10-CM	E11.3213	Type 2 diabetes mellitus with mild nonproliferative diabetic retinopathy with macular edema, bilateral	DM
ICD-10-CM	E11.649	Type 2 diabetes mellitus with hypoglycemia without coma	DM
ICD-10-CM	E11.65	Type 2 diabetes mellitus with hyperglycemia	DM
ICD-10-CM	E11.621	Type 2 diabetes mellitus with foot ulcer	DM
ICD-10-CM	E11.42	Type 2 diabetes mellitus with diabetic polyneuropathy	DM
ICD-10-CM	E11.40	Type 2 diabetes mellitus with diabetic neuropathy, unsp	DM
ICD-10-CM	E11.610	Type 2 diabetes mellitus with diabetic neuropathic arthropathy	DM
ICD-10-CM	E11.43	Type 2 diabetes mellitus with diabetic autonomic (poly)neuropathy	DM
ICD-10-CM	E11.44	Type 2 diabetes mellitus with diabetic amyotrophy	DM
ICD-10-CM	E11.3299	Type 2 diab with mild nonp rtnop without macular edema, unsp	DM
ICD-10-CM	E10.9	Type 1 diabetes mellitus without complications	DM
ICD-10-CM	E10.649	Type 1 diabetes mellitus with hypoglycemia without coma	DM
ICD-10-CM	E10.65	Type 1 diabetes mellitus with hyperglycemia	DM
ICD-10-CM	E13.44	Other specified diabetes mellitus with diabetic amyotrophy	DM
ICD-9-CM	359.1	Wielander’s distal dystrophy (HC code)	Heme/Immune
ICD-9-CM	287.2	Vasculitis, hemorrhagic	Heme/Immune
ICD-9-CM	287.5	Unspecified thrombocytopenia	Heme/Immune
ICD-9-CM	279.3	Unspecified immunity deficiency	Heme/Immune
ICD-9-CM	287.9	Unspecified hemorrhagic conditions	Heme/Immune
ICD-9-CM	279.9	Unspecified disorder of immune mechanism	Heme/Immune
ICD-9-CM	795.09	Unexplained endometrial cells on cervical Pap smear	Heme/Immune
ICD-9-CM	795.51	Tuberculin test reaction	Heme/Immune
ICD-10-CM	D69.6	Thrombocytopenia, unspecified	Heme/Immune
ICD-10-CM	D56.3	Thalassemia minor	Heme/Immune
ICD-9-CM	795.79	Systemic lupus erythematosus inhibitor (HC code)	Heme/Immune
ICD-9-CM	203	Stage III multiple myeloma (HC code)	Neoplasms
ICD-10-CM	D57.3	Sickle-cell trait	Heme/Immune
ICD-10-CM	D75.1	Secondary polycythemia	Heme/Immune
ICD-10-CM	D86.3	Sarcoidosis of skin	Heme/Immune
ICD-9-CM	795.52	Response to cell-mediated gamma interferon antigen without active tuberculosis	Heme/Immune
ICD-10-CM	D69.1	Qualitative platelet defects	Heme/Immune
ICD-10-CM	D68.52	Prothrombin gene mutation	Heme/Immune
ICD-9-CM	279.49	Progesterone dermatitis	Heme/Immune
ICD-9-CM	795.05	Positive cervical papilloma DNA test	Neoplasms
ICD-10-CM	Z86.73	Personal history of transient ischemic attack (TIA), and cerebral infarction without residual deficits	Heme/Immune
ICD-10-CM	Z86.711	Personal history of pulmonary embolism	Heme/Immune
ICD-10-CM	Z86.718	Personal history of other venous thrombosis and embolism	Heme/Immune
ICD-10-CM	Z86.59	Personal history of other mental and behavioral disorders	Heme/Immune
ICD-10-CM	Z92.89	Personal history of other medical treatment	Heme/Immune
ICD-10-CM	Z86.19	Personal history of other infectious and parasitic diseases	Heme/Immune
ICD-10-CM	Z86.69	Personal history of other diseases of the nervous system and sense organs	Heme/Immune
ICD-10-CM	Z86.79	Personal history of other diseases of the circulatory system	Heme/Immune
ICD-10-CM	Z86.018	Personal history of other benign neoplasm	Neoplasms
ICD-10-CM	Z86.13	Personal history of malaria	Heme/Immune
ICD-10-CM	Z86.008	Personal history of in-situ neoplasm of other site	Heme/Immune
ICD-10-CM	Z86.39	Personal history of endo, nutritional and metabolic disease	Heme/Immune
ICD-10-CM	Z86.2	Personal history of diseases of the blood and blood-forming organs and certain disorders involving the immune mechanism	Heme/Immune
ICD-10-CM	Z86.010	Personal history of colonic polyps	Heme/Immune
ICD-10-CM	Z92.21	Personal history of antineoplastic chemotherapy	Heme/Immune
ICD-9-CM	795.03	Papanicolaou smear of cervix with low grade squamous intraepithelial lesion (LGSIL)	Heme/Immune
ICD-9-CM	795.01	Papanicolaou smear of cervix with atypical squamous cells of undetermined significance (ASC-US)	Heme/Immune
ICD-9-CM	795	Papanicolaou smear - nonspecific abnormality	Heme/Immune
ICD-10-CM	D89.89	Other specified disorders involving the immune mechanism, not elsewhere classified	Heme/Immune
ICD-10-CM	D64.89	Other specified anemias	Heme/Immune
ICD-10-CM	D68.59	Other primary thrombophilia	Heme/Immune
ICD-10-CM	D61.818	Other pancytopenia	Heme/Immune
ICD-10-CM	D69.2	Other nonthrombocytopenic purpura	Heme/Immune
ICD-10-CM	D50.8	Other iron deficiency anemias	Heme/Immune
ICD-10-CM	D70.9	Neutropenia, unspecified	Heme/Immune
ICD-10-CM	D70.3	Neutropenia due to infection	Heme/Immune
ICD-10-CM	D50.9	Iron deficiency anemia, unspecified	Heme/Immune
ICD-10-CM	D50.0	Iron deficiency anemia secondary to blood loss (chronic)	Heme/Immune
ICD-10-CM	D84.9	Immunodeficiency, unspecified	Heme/Immune
ICD-10-CM	D89.42	Idiopathic mast cell activation syndrome	Heme/Immune
ICD-10-CM	D69.9	Hemorrhagic condition, unspecified	Heme/Immune
ICD-10-CM	D72.0	Genetic anomalies of leukocytes	Heme/Immune
ICD-10-CM	Z51.81	Encounter for therapeutic drug level monitoring	Heme/Immune
ICD-10-CM	Z51.5	Encounter for palliative care	Heme/Immune
ICD-10-CM	Z51.89	Encounter for other specified aftercare	Heme/Immune
ICD-10-CM	Z51.12	Encounter for antineoplastic immunotherapy	Heme/Immune
ICD-10-CM	Z51.11	Encounter for antineoplastic chemotherapy	Heme/Immune
ICD-10-CM	D72.829	Elevated white blood cell count, unspecified	Heme/Immune
ICD-10-CM	D72.9	Disorder of white blood cells, unspecified	Heme/Immune
ICD-10-CM	D89.9	Disorder involving the immune mechanism, unspecified	Heme/Immune
ICD-10-CM	D75.9	Disease of blood and blood-forming organs, unspecified	Heme/Immune
ICD-10-CM	D72.819	Decreased white blood cell count, unspecified	Heme/Immune
ICD-10-CM	D68.9	Coagulation defect, unspecified	Heme/Immune
ICD-10-CM	D64.9	Anemia, unspecified	Heme/Immune
ICD-10-CM	D68.51	Activated protein C resistance	Heme/Immune
ICD-9-CM	V65.5	Worried well	HIV
ICD-9-CM	V65.3	Weight loss, intentional	HIV
ICD-9-CM	V65.40	Visit for counseling	HIV
ICD-9-CM	V08	Viruses, lymphadenopathy-associated	HIV
ICD-9-CM	V65.49	Surgical counseling visit	HIV
ICD-9-CM	V65.8	Reasons for seeking consultation	HIV
ICD-10-CM	B20	Human immunodeficiency virus (HIV) disease	HIV
ICD-9-CM	V65.41	Exercise counseling	HIV
ICD-10-CM	Z21	Asymptomatic human immunodeficiency virus infection status	HIV
ICD-9-CM	186.9	Yolk Sac Tumour	Neoplasms
ICD-9-CM	193	Wuchernde struma langhans	Neoplasms
ICD-9-CM	189	WT (Wilms tumor) (HC code)	Neoplasms
ICD-9-CM	210.2	Warthin’s tumour	Neoplasms
ICD-10-CM	C88.0	Waldenstrom macroglobulinemia	Neoplasms
ICD-9-CM	184.4	Vulvar malignant neoplasm (HC code)	Neoplasms
ICD-9-CM	182	Uterus neoplasms	Neoplasms
ICD-9-CM	188.9	Urinary bladder cancer (HC code)	Neoplasms
ICD-10-CM	C44.90	Unspecified malignant neoplasm of skin, unspecified	Neoplasms
ICD-9-CM	173.9	Unspecified malignant neoplasm of skin, site unspecified	Neoplasms
ICD-10-CM	C44.40	Unspecified malignant neoplasm of skin of scalp and neck	Neoplasms
ICD-9-CM	173.4	Unspecified malignant neoplasm of scalp and skin of neck	Neoplasms
ICD-9-CM	201.9	Unspecified Hodgkin’s disease, site unspecified, extranodal and solid organ sites	Neoplasms
ICD-9-CM	162.9	Undifferentiated carcinoma of lung (HC code)	Neoplasms
ICD-9-CM	173.91	Ulcers, rodent	Neoplasms
ICD-9-CM	174.9	Tubular carcinoma of breast (HC code)	Neoplasms
ICD-9-CM	185	Transitional cell carcinoma of prostate (HC code)	Neoplasms
ICD-9-CM	145.9	The mouth cancers	Neoplasms
ICD-9-CM	162.3	Syndromes, Pancoast’s	Neoplasms
ICD-9-CM	173.99	Sweat gland tumor, malignant	Neoplasms
ICD-9-CM	172.9	Superficial spreading melanoma (HC code)	Neoplasms
ICD-10-CM	D25.2	Subserosal leiomyoma of uterus	Neoplasms
ICD-9-CM	154.1	Stage IV carcinoma of rectum (HC code)	Neoplasms
ICD-9-CM	210.4	Squamous papilloma of uvula	Neoplasms
ICD-9-CM	173.62	Squamous cell skin cancer, wrist	Neoplasms
ICD-9-CM	173.42	Squamous cell skin cancer, parietal	Neoplasms
ICD-9-CM	173.92	Squamous cell skin cancer	Neoplasms
ICD-9-CM	173.9	Squamous Cell Epithelioma	Neoplasms
ICD-10-CM	C44.92	Squamous cell carcinoma of skin, unspecified	Neoplasms
ICD-10-CM	C44.42	Squamous cell carcinoma of skin of scalp and neck	Neoplasms
ICD-10-CM	C44.622	Squamous cell carcinoma of skin of right upper limb, including shoulder	Neoplasms
ICD-10-CM	C44.329	Squamous cell carcinoma of skin of other parts of face	Neoplasms
ICD-10-CM	C44.321	Squamous cell carcinoma of skin of nose	Neoplasms
ICD-9-CM	154.3	Squamous cell carcinoma of anus (HC code)	Neoplasms
ICD-9-CM	173.41	Skin cancer of scalp or skin of neck	Neoplasms
ICD-9-CM	173.31	Skin cancer of nose	Neoplasms
ICD-9-CM	173.51	Skin cancer of anterior chest	Neoplasms
ICD-9-CM	197	Secondary teratoma of lung (HC code)	Neoplasms
ICD-10-CM	C78.00	Secondary malignant neoplasm of unspecified lung	Neoplasms
ICD-10-CM	C78.01	Secondary malignant neoplasm of right lung	Neoplasms
ICD-10-CM	C78.02	Secondary malignant neoplasm of left lung	Neoplasms
ICD-9-CM	154	Recurrent squamous cell carcinoma of colorectal region (HC code)	Neoplasms
ICD-9-CM	173.61	Recurrent basal cell carcinoma of shoulder	Neoplasms
ICD-9-CM	174.2	Primary malignant neoplasm of upper inner quadrant of female breast (HC code)	Neoplasms
ICD-10-CM	C44.99	Other specified malignant neoplasm of skin, unspecified	Neoplasms
ICD-10-CM	D26.9	Other benign neoplasm of uterus, unspecified	Neoplasms
ICD-10-CM	D23.9	Other benign neoplasm of skin, unspecified	Neoplasms
ICD-10-CM	D23.60	Other benign neoplasm of skin of unspecified upper limb, including shoulder	Neoplasms
ICD-10-CM	D23.30	Other benign neoplasm of skin of unspecified part of face	Neoplasms
ICD-10-CM	D23.70	Other benign neoplasm of skin of unspecified lower limb, including hip	Neoplasms
ICD-10-CM	D23.5	Other benign neoplasm of skin of trunk	Neoplasms
ICD-10-CM	D23.4	Other benign neoplasm of skin of scalp and neck	Neoplasms
ICD-10-CM	D23.71	Other benign neoplasm of skin of right lower limb, including hip	Neoplasms
ICD-10-CM	D23.62	Other benign neoplasm of skin of left upper limb, including shoulder	Neoplasms
ICD-9-CM	210	Neurofibroma of lip	Neoplasms
ICD-10-CM	D49.89	Neoplasm of unspecified behavior of other specified sites	Neoplasms
ICD-10-CM	D49.7	Neoplasm of unspecified behavior of endocrine glands and other parts of nervous system	Neoplasms
ICD-10-CM	D49.0	Neoplasm of unspecified behavior of digestive system	Neoplasms
ICD-10-CM	D49.2	Neoplasm of unspecified behavior of bone, soft tissue, and skin	Neoplasms
ICD-10-CM	D48.9	Neoplasm of uncertain behavior, unspecified	Neoplasms
ICD-10-CM	D48.5	Neoplasm of uncertain behavior of skin	Neoplasms
ICD-9-CM	172.6	Nail bed melanoma, upper extremity (HC code)	Neoplasms
ICD-10-CM	C90.00	Multiple myeloma not having achieved remission	Neoplasms
ICD-10-CM	D47.2	Monoclonal gammopathy	Neoplasms
ICD-9-CM	172.5	Melanoma, skin of trunk	Neoplasms
ICD-9-CM	172.7	Melanoma, skin of lower limb	Neoplasms
ICD-10-CM	D03.4	Melanoma in situ of scalp and neck	Neoplasms
ICD-10-CM	D03.59	Melanoma in situ of other part of trunk	Neoplasms
ICD-10-CM	D03.62	Melanoma in situ of left upper limb, including shoulder	Neoplasms
ICD-10-CM	D22.9	Melanocytic nevi, unspecified	Neoplasms
ICD-10-CM	D22.70	Melanocytic nevi of unspecified lower limb, including hip	Neoplasms
ICD-10-CM	D22.60	Melanocytic nevi of unsp upper limb, including shoulder	Neoplasms
ICD-10-CM	D22.5	Melanocytic nevi of trunk	Neoplasms
ICD-10-CM	D22.4	Melanocytic nevi of scalp and neck	Neoplasms
ICD-10-CM	D22.61	Melanocytic nevi of right upper limb, including shoulder	Neoplasms
ICD-10-CM	D22.71	Melanocytic nevi of right lower limb, including hip	Neoplasms
ICD-10-CM	D22.39	Melanocytic nevi of other parts of face	Neoplasms
ICD-10-CM	D22.62	Melanocytic nevi of left upper limb, including shoulder	Neoplasms
ICD-10-CM	D22.72	Melanocytic nevi of left lower limb, including hip	Neoplasms
ICD-10-CM	C51.9	Malignant neoplasm of vulva, unspecified	Neoplasms
ICD-10-CM	C55	Malignant neoplasm of uterus, part unspecified	Neoplasms
ICD-9-CM	174.4	Malignant neoplasm of upper-outer quadrant of right female breast (HC code)	Neoplasms
ICD-10-CM	C50.412	Malignant neoplasm of upper-outer quadrant of left female breast	Neoplasms
ICD-10-CM	C50.211	Malignant neoplasm of upper-inner quadrant of right female breast	Neoplasms
ICD-10-CM	C34.12	Malignant neoplasm of upper lobe, left bronchus or lung	Neoplasms
ICD-10-CM	C50.919	Malignant neoplasm of unspecified site of unspecified female breast	Neoplasms
ICD-10-CM	C50.912	Malignant neoplasm of unspecified site of left female breast	Neoplasms
ICD-10-CM	C34.90	Malignant neoplasm of unspecified part of unspecified bronchus or lung	Neoplasms
ICD-10-CM	C34.92	Malignant neoplasm of unspecified part of left bronchus or lung	Neoplasms
ICD-10-CM	C64.9	Malignant neoplasm of unspecified kidney, except renal pelvis	Neoplasms
ICD-10-CM	C50.911	Malignant neoplasm of unsp site of right female breast	Neoplasms
ICD-10-CM	C73	Malignant neoplasm of thyroid gland	Neoplasms
ICD-10-CM	C64.1	Malignant neoplasm of right kidney, except renal pelvis	Neoplasms
ICD-10-CM	C20	Malignant neoplasm of rectum	Neoplasms
ICD-10-CM	C19	Malignant neoplasm of rectosigmoid junction	Neoplasms
ICD-10-CM	C61	Malignant neoplasm of prostate	Neoplasms
ICD-10-CM	C50.819	Malignant neoplasm of overlapping sites of unspecified female breast	Neoplasms
ICD-10-CM	C50.012	Malignant neoplasm of nipple and areola, left female breast	Neoplasms
ICD-10-CM	C06.9	Malignant neoplasm of mouth, unspecified	Neoplasms
ICD-10-CM	C34.31	Malignant neoplasm of lower lobe, right bronchus or lung	Neoplasms
ICD-10-CM	C64.2	Malignant neoplasm of left kidney, except renal pelvis	Neoplasms
ICD-10-CM	C54.1	Malignant neoplasm of endometrium	Neoplasms
ICD-10-CM	C62.11	Malignant neoplasm of descended right testis	Neoplasms
ICD-10-CM	C50.111	Malignant neoplasm of central portion of right female breast	Neoplasms
ICD-10-CM	C67.9	Malignant neoplasm of bladder, unspecified	Neoplasms
ICD-10-CM	C21.0	Malignant neoplasm of anus, unspecified	Neoplasms
ICD-10-CM	C43.9	Malignant melanoma of skin, unspecified	Neoplasms
ICD-10-CM	C80.1	Malignant (primary) neoplasm, unspecified	Neoplasms
ICD-10-CM	C62.91	Malig neoplm of right testis, unsp descended or undescended	Neoplasms
ICD-10-CM	C62.90	Malig neoplasm of unsp testis, unsp descended or undescended	Neoplasms
ICD-10-CM	D25.9	Leiomyoma of uterus, unspecified	Neoplasms
ICD-10-CM	D25.1	Intramural leiomyoma of uterus	Neoplasms
ICD-10-CM	D05.10	Intraductal carcinoma in situ of unspecified breast	Neoplasms
ICD-10-CM	C81.90	Hodgkin lymphoma, unspecified, unspecified site	Neoplasms
ICD-10-CM	D18.00	Hemangioma unspecified site	Neoplasms
ICD-10-CM	D18.01	Hemangioma of skin and subcutaneous tissue	Neoplasms
ICD-10-CM	D47.3	Essential (hemorrhagic) thrombocythemia	Neoplasms
ICD-10-CM	D09.9	Carcinoma in situ, unspecified	Neoplasms
ICD-10-CM	D04.39	Carcinoma in situ of skin of other parts of face	Neoplasms
ICD-10-CM	D01.3	Carcinoma in situ of anus and anal canal	Neoplasms
ICD-10-CM	D36.9	Benign neoplasm, unspecified site	Neoplasms
ICD-10-CM	D27.9	Benign neoplasm of unspecified ovary	Neoplasms
ICD-10-CM	D31.30	Benign neoplasm of unspecified choroid	Neoplasms
ICD-10-CM	D12.3	Benign neoplasm of transverse colon	Neoplasms
ICD-10-CM	D24.1	Benign neoplasm of right breast	Neoplasms
ICD-10-CM	D36.10	Benign neoplasm of peripheral nerves and autonomic nervous system, unspecified	Neoplasms
ICD-10-CM	D36.13	Benign neoplasm of peripheral nerves and autonomic nervous system of lower limb, including hip	Neoplasms
ICD-10-CM	D11.0	Benign neoplasm of parotid gland	Neoplasms
ICD-10-CM	D36.7	Benign neoplasm of other specified sites	Neoplasms
ICD-10-CM	D10.39	Benign neoplasm of other parts of mouth	Neoplasms
ICD-10-CM	D32.9	Benign neoplasm of meninges, unspecified	Neoplasms
ICD-10-CM	D11.9	Benign neoplasm of major salivary gland, unspecified	Neoplasms
ICD-10-CM	D24.2	Benign neoplasm of left breast	Neoplasms
ICD-10-CM	D13.7	Benign neoplasm of endocrine pancreas	Neoplasms
ICD-10-CM	D33.3	Benign neoplasm of cranial nerves	Neoplasms
ICD-10-CM	D21.9	Benign neoplasm of connective and other soft tissue, unspecified	Neoplasms
ICD-10-CM	D21.10	Benign neoplasm of connective and other soft tissue of unspecified upper limb, including shoulder	Neoplasms
ICD-10-CM	D12.6	Benign neoplasm of colon, unspecified	Neoplasms
ICD-10-CM	D16.4	Benign neoplasm of bones of skull and face	Neoplasms
ICD-10-CM	D17.9	Benign lipomatous neoplasm, unspecified	Neoplasms
ICD-10-CM	D17.20	Benign lipomatous neoplasm of skin and subcutaneous tissue of unspecified limb	Neoplasms
ICD-10-CM	D17.23	Benign lipomatous neoplasm of skin and subcutaneous tissue of right leg	Neoplasms
ICD-10-CM	D17.21	Benign lipomatous neoplasm of skin and subcutaneous tissue of right arm	Neoplasms
ICD-10-CM	D17.24	Benign lipomatous neoplasm of skin and subcutaneous tissue of left leg	Neoplasms
ICD-10-CM	D17.22	Benign lipomatous neoplasm of skin and subcutaneous tissue of left arm	Neoplasms
ICD-10-CM	D17.0	Benign lipomatous neoplasm of skin and subcutaneous tissue of head, face and neck	Neoplasms
ICD-10-CM	C44.611	Basal cell carcinoma skin/ unsp upper limb, inc shoulder	Neoplasms
ICD-10-CM	C44.91	Basal cell carcinoma of skin, unspecified	Neoplasms
ICD-10-CM	C44.41	Basal cell carcinoma of skin of scalp and neck	Neoplasms
ICD-10-CM	C44.319	Basal cell carcinoma of skin of other parts of face	Neoplasms
ICD-10-CM	C44.519	Basal cell carcinoma of skin of other part of trunk	Neoplasms
ICD-10-CM	C44.311	Basal cell carcinoma of skin of nose	Neoplasms
ICD-10-CM	Z34.81	Encounter for suprvsn of normal pregnancy, first trimester	Pregnancy
ICD-10-CM	Z34.80	Encounter for supervision of other normal pregnancy, unspecified trimester	Pregnancy
ICD-10-CM	Z34.83	Encounter for supervision of other normal pregnancy, third trimester	Pregnancy
ICD-10-CM	Z34.82	Encounter for supervision of other normal pregnancy, second trimester	Pregnancy
ICD-10-CM	Z34.90	Encounter for supervision of normal pregnancy, unspecified, unspecified trimester	Pregnancy
ICD-10-CM	Z34.93	Encounter for supervision of normal pregnancy, unspecified, third trimester	Pregnancy
ICD-10-CM	Z34.91	Encounter for supervision of normal pregnancy, unspecified, first trimester	Pregnancy
ICD-10-CM	Z34.00	Encounter for supervision of normal first pregnancy, unspecified trimester	Pregnancy
ICD-10-CM	Z34.03	Encounter for supervision of normal first pregnancy, third trimester	Pregnancy
ICD-10-CM	Z34.02	Encounter for supervision of normal first pregnancy, second trimester	Pregnancy
ICD-10-CM	Z34.01	Encounter for supervision of normal first pregnancy, first trimester	Pregnancy
ICD-10-CM	Z32.00	Encounter for pregnancy test, result unknown	Pregnancy
ICD-10-CM	Z32.01	Encounter for pregnancy test, result positive	Pregnancy
